# Benchmarking readability, reliability, and scientific quality of large language models in communicating organoid science

**DOI:** 10.3389/fbioe.2026.1750225

**Published:** 2026-01-16

**Authors:** Man Sun, Dan Zang, Jun Chen

**Affiliations:** Department of Oncology, The Second Hospital of Dalian Medical University, Dalian, Liaoning, China

**Keywords:** artificial intelligence, large language models, online medical information, organoids, readability

## Abstract

**Background:**

Organoids have become central platforms in precision oncology and translational research, increasing the need for communication that is accurate, transparent, and clinically responsible. Large language models (LLMs) are now widely consulted for organoid-related explanations, but their ability to balance readability, scientific rigor, and educational suitability has not been systematically established.

**Methods:**

Five mainstream LLMs (GPT-5, DeepSeek, Doubao, Tongyi Qianwen, and Wenxin Yiyan) were systematically evaluated using a curated set of thirty representative organoid-related questions. For each model, twenty outputs were independently scored using the C-PEMAT-P scale, the Global Quality Score (GQS), and seven validated readability indices. Between-model differences were analyzed using one-way ANOVA or Kruskal–Wallis tests, and correlation analyses were performed to examine associations between readability and quality measures.

**Results:**

Model performance differed markedly, with GPT-5 achieving the highest C-PEMAT and GQS scores (16.05 ± 1.10; 4.70 ± 0.47; both P < 0.001), followed by intermediate performance from DeepSeek and Doubao (C-PEMAT 11.75 ± 2.07 and 12.05 ± 1.82; GQS 3.65 ± 0.49 and 3.35 ± 0.49). Tongyi Qianwen and Wenxin Yiyan comprised the lowest-performing tier (C-PEMAT 7.85 ± 1.09 and 9.00 ± 2.05; GQS 1.55 ± 0.51 and 2.10 ± 0.55). Score-distribution patterns further highlighted reliability gaps, with GPT-5 showing tightly clustered values and domestic models displaying broader dispersion and unstable performance. Readability differed significantly across models and question categories, with safety-related, diagnostic, and technical questions showing the highest linguistic and conceptual complexity. Correlation analyses showed strong internal coherence among readability indices but only weak-to-moderate associations with C-PEMAT, GQS, and reliability metrics, indicating that linguistic simplicity is not a dependable surrogate for scientific quality.

**Conclusion:**

LLMs exhibited substantial variability in communicating organoid-related information, forming distinct performance tiers with direct implications for patient education and translational decision-making. Because readability, scientific quality, and reliability diverged across models, linguistic simplification alone is insufficient to guarantee accurate or dependable interpretation. These findings underscore the need for organoid-adapted AI systems that integrate domain-specific knowledge, convey uncertainty transparently, ensure output reliability, and safeguard safety-critical information.

## 1 Introduction

Organoid technology has rapidly evolved into a core platform in modern bioengineering and translational oncology, providing three-dimensional systems that more faithfully recapitulate human tissue architecture, lineage dynamics and treatment response than traditional models (Xia et al., 2019; Verstegen et al., 2025). These models now support a broad array of precision-oncology applications, including drug screening, toxicity evaluation, host–microbe interaction research, and early-phase therapeutic development (Abdel-Rehim et al., 2025). As their use expands from specialized laboratories to multi-center translational pipelines and early clinical testing, the demand for communication that is accurate, accessible, and contextualized to experimental and clinical realities has intensified (Wang et al., 2024; Wang D. et al., 2025). Yet organoid science remains conceptually complex and operationally heterogeneous, creating persistent challenges for users who often turn to online resources to navigate this rapidly advancing field.

Large language models (LLMs) now function as major intermediaries in biomedical communication and are increasingly consulted for organoid-related information, from basic definitions to culture systems, drug-testing workflows and safety considerations (Chen et al., 2025; Sandmann et al., 2025). Their ability to deliver fluent, structured, and seemingly authoritative explanations positions them as promising tools for bridging knowledge gaps among diverse user groups. Yet studies in oncology, rheumatology, and dermatology show that LLMs often fail to balance mechanistic accuracy with appropriate uncertainty disclosure and safety framing (Venerito et al., 2023). They may overlook key distinctions between organoids and genetic testing, downplay sampling risks, misrepresent predictive validity, or omit essential caveats related to assay limitations (Jensen and Little, 2023). Furthermore, LLM outputs can vary substantially across prompts, sessions, and domains, raising concerns not only about accuracy but also about the reliability and stability of generated explanations—an issue of particular relevance in organoid science, where misunderstandings regarding culture conditions, lineage stability, translational readiness, or discordant results may shape experimental decisions, therapeutic choices, financial planning, and patient expectations (Puschhof et al., 2021; Wang Q. et al., 2025).

Although LLMs are increasingly incorporated into laboratory workflows, clinical counselling, and public-facing biomedical communication, their performance specific to organoid science remains poorly characterized. Key uncertainties persist: whether LLMs can accurately articulate core biological principles such as niche dependence and self-organization; whether they provide appropriately cautious interpretations of drug-response data, hereditary risk, and safety considerations; whether their outputs are consistent and reliable across similar queries; whether they correctly frame the clinical utility, turnaround time, and financial aspects of organoid testing; and whether their language is sufficiently readable and actionable for users with diverse scientific literacy levels (Grippaudo et al., 2024; Steyvers et al., 2025). In the absence of systematic evaluation, the safety and reliability of LLM-mediated organoid communication cannot be assured.

To fill this gap, we conducted a systematic, multi-dimensional benchmarking analysis of five widely used LLMs using thirty representative organoid-related questions spanning five practical domains: Technical Cognition, Diagnostic and Therapeutic Value, Safety Concerns, Cost and Process, and Decision Reference. Model outputs were assessed using validated patient-education suitability metrics (C-PEMAT-P), a global scientific quality score, and seven established readability indices, alongside inter-rater consistency measures that enabled evaluation of output reliability (Tam et al., 2024). This framework allowed us to disentangle intrinsic model-level performance differences from the domain-specific communication challenges inherent to organoid-related information.

By mapping how contemporary LLMs interpret, simplify, and at times distort organoid science, this study delivers the first comprehensive and evidence-based evaluation of AI-mediated communication in this high-complexity biomedical domain (Liu Y. et al., 2025). Importantly, it reveals that readability, scientific quality, and reliability diverge substantially across models, highlighting where LLMs can responsibly contribute to knowledge dissemination, where they introduce risks requiring caution, and how next-generation domain-adapted systems and governance frameworks should be constructed to align AI-generated explanations with the conceptual, ethical, and translational demands of organoid research, clinical decision-making, and public communication. Based on differences in model architecture, training strategies, and domain exposure, we hypothesized that large language models would exhibit systematic performance stratification rather than uniform capability in communicating organoid-related concepts. We further anticipated that linguistic readability would not align consistently with scientific quality or reliability, reflecting a structural dissociation between surface accessibility and mechanistic fidelity. Finally, we expected that questions involving safety considerations and therapeutic interpretation would pose greater challenges than descriptive or logistical queries, given their reliance on multi-step reasoning, uncertainty handling, and clinically bounded inference.

## 2 Materials and methods

### 2.1 Ethical considerations

All data used in this study were generated by LLMs and did not involve human participants, patient-identifiable information, biological specimens, or animal experiments. No content was obtained from clinical records, and no interventions were performed. In accordance with institutional and international academic standards, research based solely on publicly accessible AI-generated data does not require ethical approval.

### 2.2 Research procedure

Three specialists in organoid biology and translational oncology designed a structured set of 30 representative questions to capture the practical information needs surrounding organoid technology. Question development was informed by authoritative literature, laboratory training materials, and recurrent inquiries from patients, clinicians, and early-career researchers. After several rounds of refinement, the questions were consolidated and classified into five domains: Technical Cognition, Diagnostic and Therapeutic Value, Safety Concerns, Cost and Process, and Decision Reference, as shown in Table 1. Each question was then submitted verbatim to five widely accessible LLMs within a fixed 2-day period. To approximate real-world user behavior, a new session was initiated for each query, and no follow-up prompts, clarifications, or optimization strategies were provided. When multiple responses were generated, the first complete answer was selected. This approach was chosen to approximate typical real-world user interactions, in which only the initial response is usually consulted, while acknowledging that alternative sampling strategies could capture within-model variability. All outputs were compiled into a standardized dataset, anonymized, and assigned randomized identifiers; any metadata that could reveal the model’s identity was removed prior to evaluation. The resulting corpus served as a domain-specific benchmark that blinded expert reviewers assessed across three predefined dimensions: readability, reliability, and scientific quality. The five large language models evaluated in this study were selected because they are among the most widely accessible and commonly consulted systems for biomedical information in real-world settings, collectively representing both internationally deployed and regionally dominant platforms. This selection was intended to prioritize ecological validity and generalizability, enabling a focused assessment of how commonly used LLMs communicate organoid-related concepts in practice.

**TABLE 1:** Issue list.

Issue list
I. Technical cognition
1. What is the definition of an organoid? Can it simulate the responses of human organs (e.g., can liver organoids be used to test drug efficacy for patients with liver diseases)?
2. Is it necessary to use the patient’s own cells for organoid culture? Is the pain level of sampling comparable to that of a biopsy?
3. What is the core difference between organoid technology and genetic testing? Does organoid technology fall into the category of genetic testing?
4. Are drugs or chemical reagents used in organoid culture? Will they affect the accuracy of test results?
II. Diagnostic and therapeutic value
1. Can organoids help screen effective drugs for patients with poor treatment outcomes? Is it only used for diagnosis or can it also optimize treatment plans?
2. When early-stage cancer patients receive conventional treatment plans, is it necessary to introduce organoids? Can it reduce the risk of recurrence?
3. Can organoids predict the risk of developing familial genetic diseases? Can they assist in the prevention of genetic diseases in patients' children?
4. What are the therapeutic advantages of organoid-assisted treatment for tumor compared with traditional methods? Is the improvement in therapeutic efficacy significant?
III. Safety concerns
1. When collecting the patient’s cells for organoid culture, will it damage the patient’s organs (e.g., will collecting liver cells affect liver function)?
2. If organoids indicate that a drug is ineffective, will it cause the patient to miss effective treatment? How to avoid the risk of inaccurate testing?
3. Have patients who used organoids experienced adverse reactions? What is the occurrence probability and severity?
4. Will the patient’s cell samples and medical information be leaked to third parties? Are the privacy protection measures adequate?
IV. Cost and process
1. What is the total cost of the entire organoid testing process? Can a detailed cost breakdown be provided (e.g., sampling fee, culture fee, analysis fee)?
2. If this project is not available locally, how many round trips does the patient need to make to a provincial capital hospital? Do sampling and result collection need to be done in separate trips?
3. Is the cost of organoid testing covered by medical insurance? Can critical illness insurance cover part of the cost? What is the reimbursement ratio?
4. How long does it take to get the results of organoid testing? Can the process be expedited for patients with urgent conditions (is there an additional fee for expedited service)? How long will it take to get the results after expediting?
V. Decision reference
1. When a doctor recommends the use of organoids, is it because the patient’s condition is special or because the technology has been widely applied? Can clinical cases of similar conditions be provided?
2. When the results of organoid testing are inconsistent with those of traditional examinations, which one should be prioritized? What further examinations are needed for confirmation?
3. What is the maturity level of organoid technology? Has it passed national approval (is the safety of its clinical application guaranteed)?
4. If a patient only receives traditional treatment, will the recovery speed be slower? Is there a difference in therapeutic efficacy compared with organoid-assisted treatment? What are the specific manifestations of the difference?

### 2.3 Readability evaluation

We employed multiple formulas from the Text Readability Assessment Tool (http://readabilityformulas.com/) to quantitatively assess the readability of LLM-generated responses. Because no single optimal metric has been established and no universally accepted gold standard exists for biomedical text readability, we applied a set of widely used indices that have been consistently adopted in prior research.

The following metrics were calculated for each response (Table 2): the Coleman–Liau Index (CLI), Linear Write Index (LW), Automated Readability Index (ARI), Simple Measure of Gobbledygook (SMOG), Fog Index, Flesch Reading Ease Score (FRES), and Flesch–Kincaid Grade Level (FKGL) (Özduran and Hanci, 2022; Yilmaz Hanci, 2023; Hanci et al., 2024). Each index emphasizes distinct linguistic features, including sentence structure, word length, syllabic complexity, and lexical density, thereby justifying the use of a multi-metric approach to capture different aspects of language difficulty. These metrics capture complementary dimensions of linguistic complexity, including sentence length, word length, and lexical difficulty, and provide estimates of how closely model-generated language approximates standard written English and its comprehensibility for non-expert readers. All indices were computed on the unedited raw outputs using identical software settings to ensure objective, model-agnostic comparison. To preserve the integrity of model-generated linguistic features, no manual correction, segmentation, or text cleaning was performed.

**TABLE 2:** Readability tools, formulas and descriptions.

Readability index	Description	Formula
Gunning FOG (GFOG)	It estimates the number of years of education required for a person to understand a given text	G = 0.4 X (W/S+((C*W) X 100))
Flesch reading Ease score (FRES)	It was created to assess the readability of newspapers and is particularly effective for evaluating school textbooks and technical manuals. The scores range from 0 to 100, with higher scores indicating greater ease of reading	I = (206.835 – (84.6 X (B/W)) – (1.015 X (W/S)))
Flesch–Kincaid grade level (FKGL)	Delineates the academic capacity level imperative for grasping the written material	G = (11.8 X (B/W)) + (0.39 X (W/S)) – 15.59
Simple measure of Gobbledygook (SMOG)	It measures the number of years of education the average person needs to understand a text	G = 1.0430 X √C + 3.1291
Coleman–Liau (CL) score	Evaluates the educational level required for understanding a text and offers an associated grade level in the US education system	G = (−27.4004 X (E/100)) + 23.06395
Linsear Write (LW)	Offers an approximate assessment of the academic level needed to comprehend the text	LW = (R+3C)/S result • If > 20, divide by 2 • If ≤ 20, subtract 2, and then divide by 2
Automated readability index (ARI)	Assesses the scholastic rank in American educational institutions needed to be capable of comprehending written material. The greater the number of characters, the more complex the term	ARI = 4.71 X I+0.5*ASL – 21.43

G, Grade level; B, Number of syllables; W, Number of words; S, Number of sentences; I, Flesch index score; SMOG, Simple measure of gobbledygook; C, Complex words (≥3 syllables); E, Predicted Cloze percentage = 141.8401 – (0.214590 X number of characters) + (1.079812*S); C*, Complex words with exceptions including, proper nouns, words made 3 syllables by addition of “ed” or “es”, compound words made of simpler words. ASL, the average number of sentences per 100 words R, the number of words ≤2 syllables.

### 2.4 Reliability and quality assessment

This study used the C-PEMAT-P scale and the Global Quality Score (GQS) to evaluate the comprehensibility, actionability, and scientific quality of LLM-generated responses. Prior to formal scoring, the two evaluators underwent a calibration process using a subset of representative responses to ensure consistent interpretation of the scoring criteria. All responses were then independently scored by both reviewers. Inter-rater agreement was assessed using Cohen’s kappa coefficient, and discrepancies were resolved through discussion and adjudication by a third senior reviewer. This workflow was designed to ensure scoring consistency, reliability, and reproducibility across evaluators. The C-PEMAT-P includes 24 binary-scored items across two domains: Comprehensibility (16 items), which assesses logical structure, clarity of biological explanations, terminological accuracy, and sufficiency of background context; and Actionability (8 items), which evaluates whether the text provides specific, usable guidance, appropriately framed safety information, and content aligned with user needs (Gunduz et al., 2024). Total scores range from 0 to 24, with higher scores indicating greater suitability for patient education.

The GQS provides a global qualitative rating of content accuracy, coherence, depth, and practical relevance using a five-point scale, where scores from 1 to 5 correspond to poor, weak, moderate, good, and excellent scientific quality, respectively (Nian et al., 2024).

To assess reliability, two senior experts in organoid biology and translational medicine independently evaluated all responses. Inter-rater agreement was quantified using Cohen’s kappa coefficient, with values > 0.75 interpreted as excellent reliability (Rau and Shih, 2021). Any discrepancies were resolved through adjudication by a third senior reviewer. Both assessment tools demonstrated high inter-rater consistency, ensuring that the evaluation of LLM performance was methodologically robust and reproducible across reviewers (Faherty et al., 2020).

### 2.5 Statistical analysis

Statistical analyses were performed according to the distributional characteristics of each variable. Continuous variables that met normality requirements, including C-PEMAT-P and GQS values, were summarized as mean ± standard deviation and compared across the five LLMs using one-way analysis of variance (ANOVA) with Bonferroni-adjusted *post hoc* testing. Metrics that did not show normal distribution, such as the ARI and FRES, were summarized as median with interquartile range and analyzed using the Kruskal–Wallis H test, followed by Dunn’s *post hoc* tests with adjusted significance thresholds when applicable. All statistical tests were two-tailed, with significance defined as P < 0.05. Data analysis was conducted using IBM SPSS Statistics version 25.0, and visualizations were generated with GraphPad Prism version 9.0. All prompts and model settings used for model querying are provided in the Supplementary Material to facilitate reproducibility.

## 3 Results

### 3.1 Readability analysis

This study systematically examined how different LLMs and content categories influence the readability and overall quality of organoid-related educational text. We compared five widely used models (DeepSeek, Doubao, GPT-5, Tongyi Qianwen, and Wenxin Yiyan) across three evaluation dimensions: patient-education suitability (C-PEMAT-P), overall scientific quality (GQS), and seven established readability indices (ARI, FRES, GFOG, FKGL, CLI, SMOG, and LW). We also analyzed these metrics across five thematic domains of organoid education—Technical Cognition, Diagnostic and Therapeutic Value, Safety Concerns, Cost and Process, and Decision Reference. By integrating model-level and domain-level analyses, we identified how algorithmic characteristics and question type jointly determine the clarity and educational value of AI-generated organoid information.

At the model level (Table 3), the five LLMs demonstrated substantial variation in patient-education suitability and overall scientific quality. Both C-PEMAT-P and GQS differed highly significantly across models (F = 71.22 and 124.88; both P < 0.001). GPT-5 showed the strongest performance on both metrics (C-PEMAT 16.05 ± 1.10; GQS 4.70 ± 0.47), DeepSeek and Doubao formed an intermediate tier (C-PEMAT ∼11.75–12.05; GQS ∼3.35–3.60), and Tongyi Qianwen and Wenxin Yiyan represented the lowest-performing group (C-PEMAT 7.85 ± 1.09 and 9.00 ± 2.05; GQS 1.55 ± 0.51 and 2.10 ± 0.55). All seven readability indices also differed significantly among models (all P < 0.001). Based on median values, GPT-5 and Wenxin Yiyan generated text with higher ARI, GFOG, FKGL, CLI, and SMOG and lower FRES, indicating longer sentences, denser terminology, and greater reading difficulty. DeepSeek and Tongyi Qianwen produced more readable outputs, with Doubao falling in between. LW values also varied significantly, with GPT-5 showing the lowest and Wenxin Yiyan the highest median LW, suggesting model-specific patterns in sentence and paragraph structuring.

**TABLE 3:** Model-level analysis of readability and quality metrics.

Variables	Total (n = 100)	Deep seek (n = 20)	Doubao (n = 20)	GPT-5 (n = 20)	Tongyi Qianwen (n = 20)	Wenxin Yiyan (n = 20)	Statistic	*P*
C-PEMAT score, mean ± SD	11.34 ± 3.30	11.75 ± 2.07	12.05 ± 1.82	16.05 ± 1.10	7.85 ± 1.09	9.00 ± 2.05	F = 71.22	<0.001
GQS score, mean ± SD	3.07 ± 1.23	3.65 ± 0.49	3.35 ± 0.49	4.70 ± 0.47	1.55 ± 0.51	2.10 ± 0.55	F = 124.88	<0.001
ARI, M (Q_1_, Q_3_)	17.48 (15.53, 19.84)	15.91 (14.89,17.23)	18.13 (16.39,19.15)	21.93 (20.05,23.00)	17.79 (16.16,19.00)	15.11 (13.86,16.34)	χ^2^ = 50.57#	<0.001
FRES, M (Q_1_, Q_3_)	18.00 (6.75, 26.00)	22.00 (21.00,27.25)	16.50 (10.50,27.00)	0.00 (0.00,10.25)	15.00 (3.00,26.50)	26.00 (18.00,36.25)	χ^2^ = 40.32#	<0.001
GFOG, M (Q_1_, Q_3_)	17.30 (15.60, 19.10)	16.20 (15.75,17.15)	16.75 (15.67,17.95)	19.15 (18.33,20.30)	18.15 (15.80,20.07)	15.95 (15.28,18.57)	χ^2^ = 21.03#	<0.001
FKGL, M (Q_1_, Q_3_)	15.66 (14.25, 18.07)	14.85 (14.20,15.27)	15.99 (14.43,17.49)	20.00 (18.23,20.76)	15.72 (14.05,17.66)	14.23 (12.74,15.64)	χ^2^ = 43.44#	<0.001
CL, M (Q_1_, Q_3_)	17.66 (15.94, 19.14)	16.46 (15.22,17.00)	18.16 (16.59,19.09)	20.10 (18.75,21.17)	18.63 (16.62,19.63)	16.26 (13.89,17.80)	χ^2^ = 36.23#	<0.001
SMOG, M (Q_1_, Q_3_)	14.21 (12.86, 15.49)	13.29 (12.86,13.99)	14.31 (12.86,15.44)	17.24 (15.90,17.93)	14.22 (12.32,15.12)	13.02 (12.39,14.42)	χ^2^ = 40.84#	<0.001
LW, M (Q_1_, Q_3_)	52.00 (49.00, 55.25)	53.00 (51.75,56.50)	51.50 (48.75,54.00)	47.50 (45.00,50.00)	52.00 (49.75,55.50)	55.50 (52.75,59.25)	χ^2^ = 32.53#	<0.001

F: ANOVA, #: Kruskal-wails test; SD: Standard deviation, M: median, Q_1_: 1st quartile, Q_3_: 3rd quartile.

At the content-category level (Table 4), question topic had a pronounced influence on readability but only a minimal effect on quality scores. C-PEMAT-P and GQS showed no significant differences across the five domains (F = 0.21 and 0.04; P = 0.934 and 0.997), suggesting that educational suitability and scientific quality were generally stable regardless of whether questions focused on process, decision-making, or technical considerations. In contrast, most readability indices showed significant domain-related variation. ARI differed across domains (P = 0.033), while FRES, GFOG, FKGL, CLI, and SMOG exhibited even stronger differences (P values 0.002 to <0.001). LW was the only index without significant variation (P = 0.784). Responses addressing Diagnostic and Therapeutic Value, Safety Concerns, and Technical Cognition displayed higher ARI, GFOG, FKGL, and SMOG and lower FRES, indicating longer, more technical, and more difficult text. By comparison, questions related to Cost and Process and Decision Reference yielded relatively more readable outputs, though still not fully accessible for lay audiences. Overall, these findings indicate that readability is jointly shaped by intrinsic model characteristics and by the inherent complexity of the question category.

**TABLE 4:** Content-level analysis of readability and quality metrics.

Variables	Total (n = 100)	Cost and process (n = 20)	Decision reference (n = 20)	Diagnostic and therapeutic value (n = 20)	Safety concerns (n = 20)	Technical cognition (n = 20)	Statistic	*P*
C-PEMAT score, mean ± SD	11.34 ± 3.30	11.90 ± 3.16	11.00 ± 3.45	11.15 ± 3.54	11.35 ± 3.07	11.30 ± 3.54	F = 0.21	0.934
GQS score, mean ± SD	3.07 ± 1.23	3.00 ± 1.26	3.05 ± 1.23	3.05 ± 1.32	3.15 ± 1.14	3.10 ± 1.33	F = 0.04	0.997
ARI, M (Q_1_, Q_3_)	17.48 (15.53, 19.84)	16.23 (14.76,19.66)	17.54 (14.94,20.55)	18.20 (17.26,21.33)	16.20 (15.42,17.43)	18.24 (15.90,19.68)	χ^2^ = 10.52#	0.033
FRES, M (Q_1_, Q_3_)	18.00 (6.75, 26.00)	27.50 (20.25,40.00)	18.00 (5.25,26.50)	16.00 (2.25,18.50)	22.00 (14.00,29.25)	11.00 (2.75,20.25)	χ^2^ = 21.18#	<0.001
GFOG, M (Q_1_, Q_3_)	17.30 (15.60, 19.10)	15.85 (15.02,17.50)	17.00 (16.02,18.80)	19.15 (17.73,20.02)	15.95 (13.93,17.35)	17.55 (16.43,19.05)	χ^2^ = 22.25#	<0.001
FKGL, M (Q_1_, Q_3_)	15.66 (14.25, 18.07)	14.43 (12.25,16.40)	15.76 (14.11,18.34)	15.98 (15.63,19.54)	14.66 (13.97,16.09)	17.09 (15.02,18.27)	χ^2^ = 15.68#	0.003
CL, M (Q_1_, Q_3_)	17.66 (15.94, 19.14)	15.78 (13.27,17.89)	17.95 (15.82,20.45)	18.97 (17.87,19.93)	17.22 (15.64,17.88)	18.36 (16.84,19.23)	χ^2^ = 18.19#	0.001
SMOG, M (Q_1_, Q_3_)	14.21 (12.86, 15.49)	13.46 (11.98,14.54)	13.93 (12.91,15.52)	15.12 (14.39,16.51)	12.98 (12.46,14.32)	14.83 (13.21,15.53)	χ^2^ = 16.80#	0.002
LW, M (Q_1_, Q_3_)	52.00 (49.00, 55.25)	52.50 (49.75,57.50)	51.00 (48.00,56.50)	51.50 (48.75,55.00)	53.00 (49.50,55.00)	52.00 (48.75,53.25)	χ^2^ = 1.73#	0.784

F: ANOVA, #: Kruskal-wails test; SD: Standard deviation, M: median, Q_1_: 1st quartile, Q_3_: 3rd quartile.

### 3.2 Reliability and quality assessment

**FIGURE 1:**
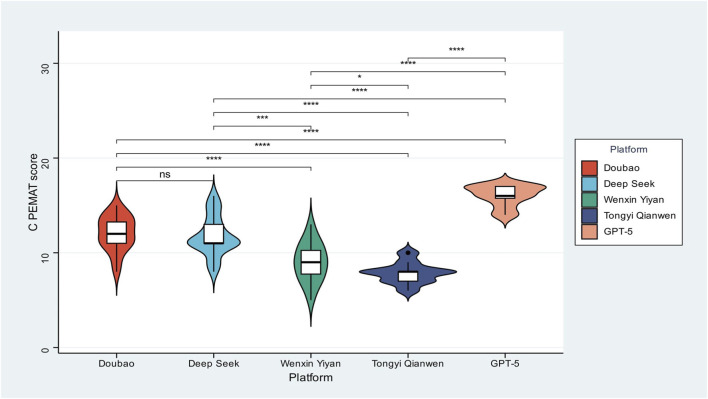
C-PEMAT scores across five large language models. Violin plots display the distribution of C-PEMAT scores for all five models. GPT-5 shows the highest and most concentrated values, indicating superior educational suitability. Doubao and DeepSeek demonstrate intermediate performance, whereas Wenxin Yiyan and Tongyi Qianwen yield consistently lower scores. Statistical significance was evaluated using one-way ANOVA followed by *post hoc* testing (*ns; *P < 0.05; **P < 0.01; ***P < 0.001; ***P < 0.0001).

Across the five models, C-PEMAT-P scores varied substantially (Figure 1), demonstrating strong model dependence in the patient-education suitability of organoid-related content and indicating considerable variability in the reliability of information provided. GPT-5 represented the highest-performing tier, with markedly higher C-PEMAT-P scores than all other models, reflecting explanations that were both cognitively accessible and operationally actionable. Doubao and DeepSeek formed a mid-level tier, with median scores above 12 and tight distribution patterns, suggesting relatively reliable and stable delivery of clear, stepwise guidance that could meaningfully support patient decision-making. In contrast, Wenxin Yiyan and Tongyi Qianwen constituted the lowest tier, with scores clustered at or below 10 and visibly broader distributions, indicating frequent production of content that is difficult to act upon, inconsistently structured, and poorly matched to patient literacy levels. This three-tier pattern suggests that LLMs differ not only in average performance but also segregate into distinct strata of educational reliability, with some domestic models approaching clinically usable clarity while others exhibit systematic limitations likely to hinder comprehension and behavioral uptake.

**FIGURE 2:**
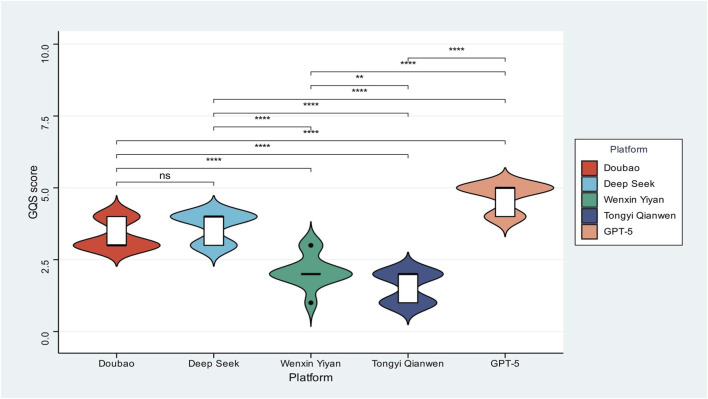
Global Quality Score distributions among five large language models. Violin plots illustrate GQS distributions across models. GPT-5 performs best, producing accurate and coherent explanations. Doubao and DeepSeek represent mid-range performers with moderate variability, while Wenxin Yiyan and Tongyi Qianwen exhibit the lowest and most dispersed scores. One-way ANOVA with *post hoc* comparisons was applied to assess significance (*ns; *P < 0.05; **P < 0.01; ***P < 0.001; ***P < 0.0001).

A similar performance hierarchy was observed for GQS (Figure 2), highlighting model architecture–related differences in scientific rigor. GPT-5 again occupied the top tier, with consistently high and tightly clustered GQS values, reflecting outputs that were accurate, coherent, and contextually appropriate in explaining organoid principles and applications. Doubao and DeepSeek formed a middle tier, characterized by overlapping medians and narrow interquartile ranges, indicating generally reliable performance but not uniformly strong adherence to evidence-based communication standards. Wenxin Yiyan and Tongyi Qianwen remained in the lowest tier, with GQS distributions skewed toward the lower end and elongated violin plots, suggesting greater variability in factual robustness and internal consistency. This graded performance pattern indicates that only a subset of current LLMs can be considered suitable for high-stakes scientific communication on organoids, whereas others may produce unstable or partially credible explanations that could compromise safe and accurate knowledge translation.

### 3.3 Correlation analysis

**FIGURE 3:**
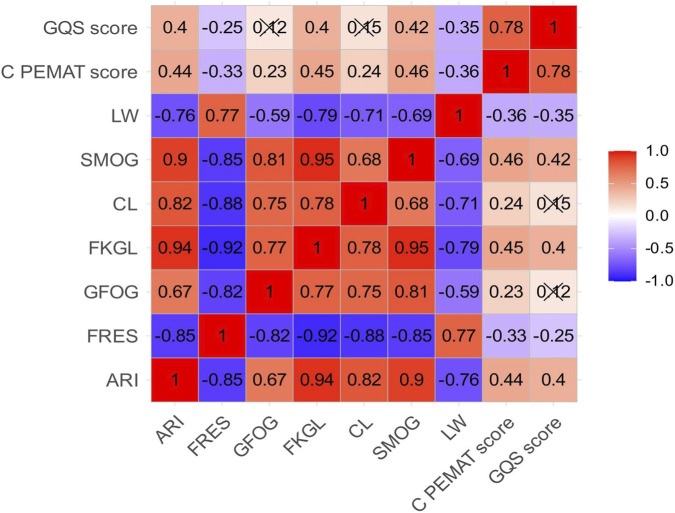
Correlation matrix of readability and quality metrics. Heatmap visualizing Pearson correlations between readability indices and quality measures. Readability metrics cluster closely, reflecting shared assessment of linguistic complexity, whereas C-PEMAT and GQS show only weak-to-moderate correlations with these indices. The pattern indicates that textual readability and scientific quality represent partially independent dimensions.

Correlation analysis showed a clear separation between readability metrics and overall text quality, although several consistent correlation patterns were identified (Figure 3). The C-PEMAT score displayed moderate correlations with several readability indicators. Positive correlations with SMOG (0.46), FKGL (0.45), ARI (0.44) and CL (0.24) indicate that higher patient-education suitability is often associated with greater lexical and syntactic complexity. Negative correlations with FRES (−0.33) and LW (−0.36) suggest that responses that are too brief or overly simplified may lack the depth or actionable detail required for effective understanding. These findings indicate that high-quality patient-education materials require a balance between clarity and informational completeness, and that the use of appropriate professional vocabulary does not reduce accessibility when embedded in clear structure and contextual explanation. The presence of moderate rather than strong correlations further suggests that readability alone cannot be used as a substitute for assessing the reliability of LLM-generated content.

In contrast, the GQS score demonstrated a different pattern of correlations. Modest positive associations with ARI (0.40), SMOG (0.42), FKGL (0.40) and CL (0.15), together with weak or negative correlations with FRES (−0.25) and LW (−0.35), indicate that higher scientific quality often involves more structured reasoning and more specialized terminology, which strengthens accuracy and coherence. The weak-to-moderate correlations show that the reliability of scientific explanations depends more on conceptual precision and internal consistency than on linguistic simplicity. Strong internal correlations among readability metrics, including ARI with FKGL (0.94), SMOG with FKGL (0.95) and SMOG with GFOG (0.81), confirm that these indicators primarily measure linguistic complexity rather than the quality of scientific reasoning. Overall, the results demonstrate that readability, quality and reliability represent partially independent dimensions. Scientific quality is shaped by accuracy, completeness and the organisation of knowledge, while readability metrics describe the linguistic form through which information is conveyed.

## 4 Discussion

Organoid technology has progressed from a specialized experimental method to a central platform in precision oncology, regenerative medicine and translational therapeutics (Zeng et al., 2024). Its conceptual complexity, protocol sensitivity and variable clinical readiness create persistent challenges for accurate communication across scientific, clinical and public settings (Getz and Campo, 2017). At the same time, LLMs have become widely used sources of organoid-related information for patients, trainees and clinicians, meaning that their strengths and limitations directly influence how this rapidly growing biotechnology is perceived (Li et al., 2025). In this context, both the quality and reliability of LLM-generated explanations are essential for maintaining scientific integrity. This study provides the first systematic and data-driven evaluation of how major LLMs communicate organoid concepts, revealing performance patterns with implications for safety, comprehension and translation (Goodman et al., 2023).

Three major insights emerge. First, model performance forms distinct tiers rather than a continuous scale. GPT-5 consistently produced the highest scores in both educational suitability and scientific quality, offering explanations that were coherent, accurate and stable. DeepSeek and Doubao constituted an intermediate tier that delivered information of acceptable structure but variable depth and inconsistent articulation of limitations. Tongyi Qianwen and Wenxin Yiyan formed a lower tier characterized by fragmented logic, missing mechanistic steps and limited educational relevance. This stratification indicates that architecture-level differences translate directly into disparities in factual stability and interpretive correctness. Because organoid communication involves sampling risk, assay interpretation and the distinction between experimental prediction and clinical judgement, using models with unstable performance may distort a knowledge base that is already technically complex and actively evolving (Gu et al., 2023; Liu X. et al., 2025). This stratification likely reflects differences in linguistic representation capacity, domain-specific biomedical exposure during training, stability of long-chain reasoning, and the depth of integrated biomedical knowledge, all of which directly shape how LLMs handle technically complex and safety-sensitive organoid-related questions.

Beyond overall score differences, the variability in reliability observed across models was associated with several recurring error patterns. First, factual inaccuracies were common in responses describing organoid capabilities or experimental constraints, often involving oversimplified or imprecise biological statements. Second, unwarranted clinical extrapolation occurred when experimental organoid findings were implicitly framed as predictive of patient-level therapeutic outcomes without appropriate qualification. Third, omission of safety-related information was frequently observed in discussions of sampling procedures, assay limitations, and downstream decision-making, reducing the completeness of risk communication. Finally, incomplete reasoning chains were evident in responses that presented locally fluent explanations but failed to maintain logical continuity across multiple inferential steps, particularly in safety- and treatment-related scenarios. Together, these patterns help explain why surface-level coherence does not necessarily translate into reliable or clinically responsible organoid communication.

Second, the readability analysis highlights a structural challenge. Although readability indices differed across models, the largest increases in linguistic and conceptual difficulty consistently appeared in responses about diagnostic and therapeutic value, safety considerations and technical principles. These categories require multi-step reasoning, mechanistic accuracy and precise terminology, all of which naturally increase reading difficulty. In comparison, questions on cost, logistics or general decision advice were somewhat easier to read yet remained non-trivial for individuals without a biomedical background. These findings suggest that expectations for uniformly simplified language are misaligned with the cognitive demands of organoid science (Di Marco et al., 2024). Moreover, excessive simplification may obscure mechanistic constraints and boundary conditions, increasing the risk of misinterpretation in technically complex contexts (Lee et al., 2025).

Third, the correlation analysis shows that readability and content quality function as partially independent dimensions. Readability indices were strongly correlated with one another but only weak to moderate in relation to C-PEMAT and GQS. Higher-quality responses tended to contain moderate lexical and syntactic complexity, reflecting the need for structured reasoning and domain-specific terminology to preserve mechanistic fidelity (Wang J. et al., 2025). In contrast, responses with very high readability often lacked the depth required to support informed decisions or meaningful understanding (Büker and Mercan, 2025). These findings demonstrate that reliability, defined as the stability, correctness and internal coherence of explanations, cannot be inferred from readability alone. Reliable communication depends on factual accuracy and explicit clarification of uncertainty rather than simplified vocabulary or shorter sentences (Magnaguagno et al., 2022). For advanced biotechnologies such as organoid systems, conceptual precision and transparent reasoning contribute more to user comprehension than reductions in linguistic complexity.

Together, these results carry practical implications. General-purpose language models should not be deployed uncritically in patient communication, laboratory onboarding or early clinical decision support. Only a subset of models demonstrates reliability adequate for safety-critical content, and reliability varies systematically by domain and query type (Ouertani et al., 2023). Communication strategies should emphasize structured and layered explanations, preserving essential terminology while adding context, stepwise logic and explicit caveats about assay limitations. Given that topics related to safety and treatment are consistently more difficult to express, additional safeguards are necessary, such as standardized descriptions of variability sources, reproducibility constraints and clear reminders that organoid assays cannot replace clinical expertise or informed consent processes (Anderson and Ledford, 2024).

From an ethical and governance perspective, responsible use of large language models in organoid communication requires the definition of minimum safeguards rather than reliance on general principles alone. Inaccurate or misleading organoid-related content generated by large language models carries distinct ethical risks. Overstated or imprecise descriptions of organoid capabilities may inflate patient expectations regarding diagnostic certainty or therapeutic benefit. In addition, misrepresentation of experimental readiness or clinical applicability may influence financial decisions, including out-of-pocket testing, participation in unproven interventions, or resource allocation. Finally, such misinformation may complicate communication between patients and clinicians by introducing unrealistic assumptions or misaligned interpretations of experimental findings, thereby undermining informed discussion and shared decision-making. First, transparency of information sources is essential, including clear indication of whether statements are derived from experimental literature, synthesized inference, or generalized biomedical knowledge. Second, model outputs should be auditable, with mechanisms to trace claims back to supporting evidence and to flag content generated under uncertainty or low confidence. Third, explicit boundaries for clinical interpretation must be enforced, ensuring that organoid-based explanations are not presented as substitutes for clinical judgment, regulatory approval, or informed consent processes. Finally, standardized safety warnings should be mandatory for topics involving tissue sampling, therapeutic interpretation, or downstream decision-making, to prevent implicit normalization of experimental findings as clinical recommendations. Together, these safeguards provide a concrete foundation for responsible AI deployment in high-risk biomedical communication contexts.

It is essential to clearly distinguish between general educational information and content that may be interpreted as personalized medical advice. Although the LLM-generated responses evaluated in this study were intended to provide general explanations of organoid concepts, topics involving drug sensitivity, disease risk, or treatment interpretation are particularly vulnerable to misinterpretation. Without explicit boundary signaling, users may construe descriptive or probabilistic statements as individualized clinical guidance, despite the absence of patient-specific data. Such unintended medical interpretation may influence treatment expectations, self-directed decision-making, and clinician–patient communication, with potentially serious implications for clinical safety and shared decision-making. These findings highlight the need for explicit disclaimers, contextual framing, and clear separation between educational explanation and individualized medical decision-making when deploying general-purpose language models in clinically adjacent domains.

The observed disparities in model performance across languages raise important fairness and equity considerations. Uneven access to high-quality, domain-specific biomedical training data across languages likely contributes to these differences, resulting in systematically lower reliability and completeness in non-dominant language contexts. Such disparities may translate into unequal access to accurate organoid-related information, disproportionately affecting patients and clinicians who rely on models operating in under-resourced languages. Addressing these gaps will require targeted investment in multilingual biomedical corpora, transparent reporting of language-specific performance, and fairness-aware evaluation frameworks to ensure equitable information access across linguistic settings. Methodologically, this study establishes a multiparametric evaluation framework that integrates educational suitability, global quality, multi-index readability profiling and correlation structures (Eweje et al., 2021). Incorporating reliability analysis further clarifies how linguistic form relates to epistemic integrity, revealing tensions that single-metric approaches cannot capture (Chen et al., 2024). This framework can be applied to other emerging biotechnologies, including CAR-based therapies, organ-on-chip systems and genome-editing platforms, where misunderstanding risk is amplified by rapid development and limited standardization (Wang H. et al., 2025). The observed performance stratification also provides a foundation for next-generation domain-adapted models, which may integrate structured ontologies, validated protocol repositories, uncertainty representations and curated translational datasets (Jain et al., 2025).

This study has several limitations. Because large language models are subject to ongoing system updates and context-dependent behavior, the findings of this study reflect model performance at a specific point in time. First, model performance was assessed at a single time point, which limits generalizability because LLMs evolve rapidly. Second, although the curated FAQ set reflects common organoid-related questions, it does not include highly specialized scenarios such as immune co-culture or lineage engineering. Third, the evaluation relied solely on textual outputs, including readability, quality and inferred reliability, without directly assessing user comprehension or decision-making. Fourth, reliability was inferred from internal consistency rather than from adversarial or uncertainty-focused testing, which may overestimate real-world robustness. Finally, the study examined general-purpose models with limited transparency in their training data, and future domain-adapted or multimodal architectures may perform differently. These considerations should inform interpretation of the findings.

Future research can advance in three interconnected directions. First, expanding model and question diversity across languages, health-literacy groups and disease-specific organoid scenarios will strengthen external validity. This expansion should be supported by multidimensional evaluation frameworks that connect objective performance metrics with user comprehension, behavioral change and clinical relevance. Incorporating behavioral endpoints will clarify how AI-generated explanations influence understanding, expectation management, adherence and shared decision-making. Second, reliability assessment should include longitudinal stability, clarity and consistency of uncertainty disclosure, cross-session reproducibility and resilience to ambiguous or clinically nuanced prompts. These elements are essential for a balanced evaluation of model quality. Third, organoid-specific models developed from curated ontologies, standardized protocols and high-quality translational datasets are needed to ensure biological accuracy. When combined with adaptive text-generation systems that adjust readability to user literacy, such models can improve the clarity and safety of organoid communication. In parallel, governance frameworks should establish minimum standards for accuracy, uncertainty disclosure, provenance transparency, reliability monitoring and long-term auditing. These efforts will support the development of safer, more interpretable and more equitable AI systems for organoid science. To advance from benchmarking to responsible deployment, future work should consolidate organoid communication into an auditable infrastructure centered on a domain-specific knowledge graph that encodes experimental constraints, translational boundaries, and clinically relevant context. This must be paired with standardized uncertainty and risk-disclosure protocols that explicitly separate experimental inference from clinical actionability in safety-critical scenarios. Embedding these mechanisms within a lifecycle governance framework with expert review and post-deployment surveillance would convert organoid-informed AI outputs from persuasive narratives into accountable scientific communication.

## 5 Conclusion

Organoid technology has become integral to precision oncology, which makes the clarity, accuracy and reliability of AI-generated explanations increasingly consequential. This study shows marked performance differences among current LLMs, and only a limited number of models can provide organoid information that is both scientifically robust and suitable for educational use. The weak correspondence between readability and scientific quality further indicates that simplified language alone cannot ensure reliable interpretation. Taken together, these findings highlight the need for organoid-adapted AI systems that integrate domain-specific knowledge, express uncertainty with clarity and support communication practices that enable safe, trustworthy and equitable translation of organoid science.

## Data Availability

The original contributions presented in the study are included in the article/Supplementary Material, further inquiries can be directed to the corresponding author.
